# Soil Resources Area Affects Herbivore Health

**DOI:** 10.3390/ijerph8062556

**Published:** 2011-06-23

**Authors:** James A. Garner, H. Anwar Ahmad, Chad M. Dacus

**Affiliations:** 1 Department of Biology, Jackson State University, Jackson, MS 39269, USA; 2 Biostatistical Support Unit, Department of Biology, Jackson State University, Jackson, MS 39217, USA; E-Mail: hafiz.a.ahmad@jsums.edu; 3 Mississippi Department of Wildlife, Fisheries, and Parks, 1505 Eastover Drive, Jackson, MS 39211, USA; E-Mail: chad.dacus@mdwfp.state.ms.us

**Keywords:** body mass, corpora lutea, fetus, kidney fat index, Mississippi, *Odocoileus virginianus*, physiographic region, soil productivity, white-tailed deer

## Abstract

Soil productivity effects nutritive quality of food plants, growth of humans and animals, and reproductive health of domestic animals. Game-range surveys sometimes poorly explained variations in wildlife populations, but classification of survey data by major soil types improved effectiveness. Our study evaluates possible health effects of lower condition and reproductive rates for wild populations of *Odocoileus virginianus* Zimmerman (white-tailed deer) in some physiographic regions of Mississippi. We analyzed condition and reproductive data for 2400 female deer from the Mississippi Department of Wildlife, Fisheries, and Parks herd health evaluations from 1991–1998. We evaluated age, body mass (Mass), kidney mass, kidney fat mass, number of corpora lutea (CL) and fetuses, as well as fetal ages. Region affected kidney fat index (KFI), which is a body condition index, and numbers of fetuses of adults (*P* ≤ 0.001). Region affected numbers of CL of adults (*P* ≤ 0.002). Mass and conception date (CD) were affected (*P* ≤ 0.001) by region which interacted significantly with age for Mass (*P* ≤ 0.001) and CD (*P* < 0.04). Soil region appears to be a major factor influencing physical characteristics of female deer.

## 1. Introduction

Many factors influence growth and productivity of wild animals, including forage nutritional quality. Sileo [1], as cited in the literature [2], reported that soil affects the number of embryos borne to *Odocoileus virginianus* Zimmerman (white-tailed deer). Small herbivores in relatively sterile coastal habitats select micro-habitats with higher dietary quality where some have grown faster [3–6].

Jacobson [7] found soil correlations to Mississippi white-tailed deer growth. Several studies in the 1980s focused on this topic on infertile barrier islands, in coastal Georgia, and the Southern Appalachian Mountains (A. S. Johnson, Emeritus, University of Georgia, personal communication). The effect has been demonstrated further in Mississippi [8] and in Texas [9,10]. In addition, fawn recruitment models differed by site productivity [11]. As a possible explanation Jones *et al*. [12] studied protein levels and hypothesized that effects may also be expressed in lactation and fawn recruitment. In contrast, minerals had no effect on antler growth [13].

There have been no studies examining the effect of soil region on all the physical variables utilized in our study. The objective of the present study was to quantify regional effects on additional physical characteristics of female deer for the temporally and spatially identical population sampled by Strickland and Demarais [14]. Their sample size was large and served as a benchmark to verify our sample as representative of the population by way of the Mass component of each dataset.

## 2. Methods

**Figure 1.**
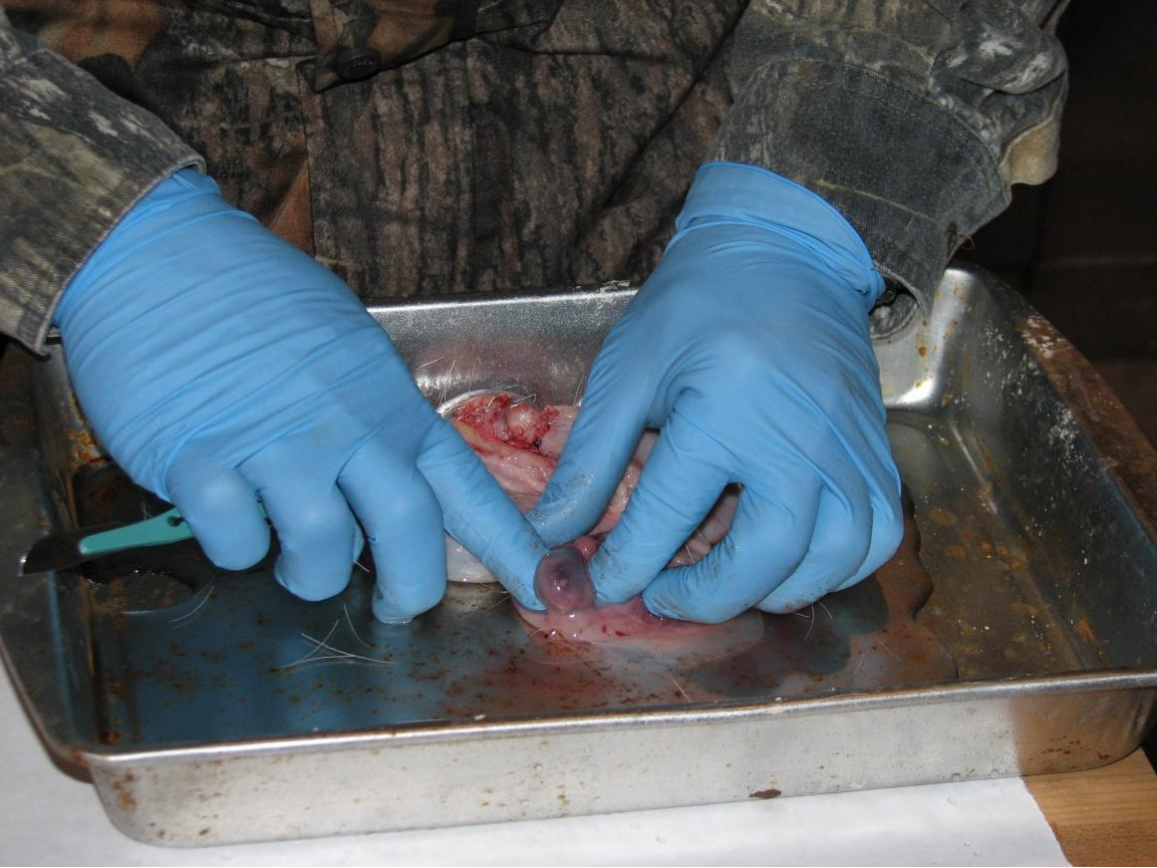
Counting corpora lutea in ovary from white-tailed deer in Mississippi.

**Figure 2.**
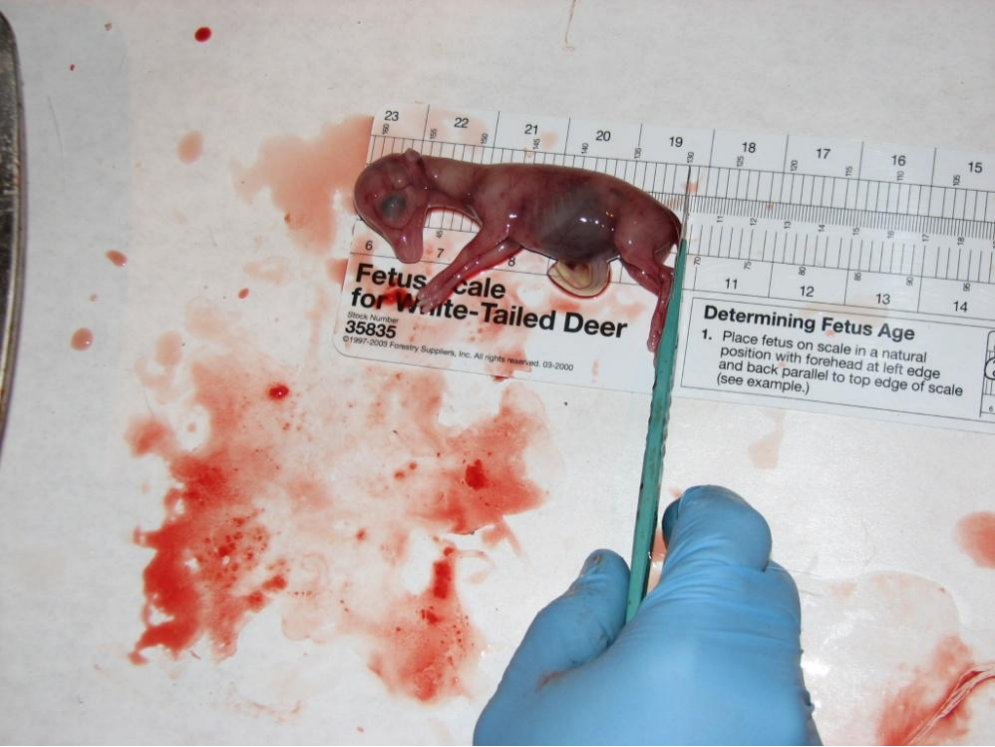
Conception date is determined from fetus length of white-tailed deer in Mississippi.

Physiology was compared across five soil regions of Mississippi. In descending order of assumed productivity, the regions are Mississippi River Valley or “Delta” (D), Loess Hills (L), Upper Coastal Plain (UCP), Lower Coastal Plain (LCP), and Coastal Flatwoods (CF). Mississippi Department of Wildlife, Fisheries, and Parks (MDWFP) personnel collected deer during January–April 1991–1998. They determined age, eviscerated Mass, numbers of fetuses and of corpora lutea (CL; Figure 1), kidney fat indices (KFI), and Julian conception date (CD; Figure 2). We grouped all deer 8.5 years old and older due to small sample sizes for these ages. We tested the effects of region, age, and age-region interactions on Mass, KFI, numbers of fetuses and CL, and CD with a 2-way, unbalanced ANOVA on 2400 females. We used SAS software for statistical analysis, specifically the General Linear Model. We used Duncan’s Multiple Range test to further classify significant means.

All deer collected were included in Mass determinations. Fetus ages were recorded only for 1.5 yr-old and older deer for CD calculations. For comparisons across regions (ages combined), only adults (2.5+-yr-olds) were analyzed for KFI, numbers of fetuses and CL in accordance with accepted management practice.

## 3. Results and Discussion

### 3.1. Body Size and Condition

Body size and condition were affected by soil region. Main effects on Mass were highly significant (P ≤ 0.001) with evident interactions (P ≤ 0.001) which suggested age-related development differed among regions and vice-versa (Table 1). Deer were heaviest from D, followed by L, then UCP, and each of those regions was greater than LCP and CF (P ≤ 0.05). The increase in Mass across those regions was 2%, 19%, 7%, and 11% from CF-LCP, LCP-UCP, UCP-L, and L-D, respectively, for an overall increase from CF-D of 46%.

**Figure 3.**
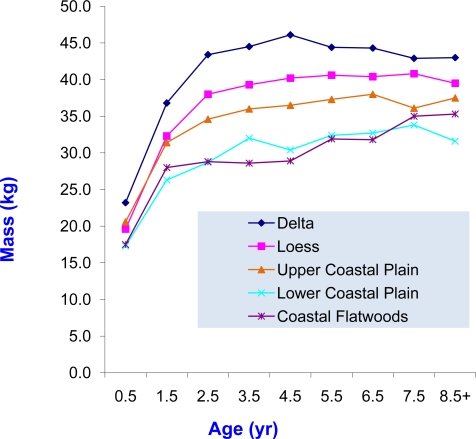
Female white-tailed deer body mass by soils in Mississippi herd health study during March 1991–1998.

Due to the soil-age interaction, Mass was presented across ages by soils (Figure 3). For most years, there were no statistical differences (P > 0.05) among soil regions, but lowest values (P ≤ 0.05) did occur for two years: in LCP (8.5+-yr-olds) and CF (3.5-yr-olds). A pattern of lower Mass from either the LCP and/or CF appears obvious. All 0.5 year-old deer were collected as a result of being mistaken for an older animal. Consequently, their mean Mass is probably biased upward; therefore, Mass from Figure 3 should not be taken as an estimate of fawn Mass for the population. Since the bias would occur in all soil regions, the pattern seen in our data across the regions would be expected to be representative of the population.

Main effects on KFI were significant (P ≤ 0.001), but not interactions (P = 0.11). KFI declined from D > L = UCP > LCP = CF with all denoted differences being significant (P ≤ 0.05). The increases in KFI across those regions were 3%, 82%, 18%, and 21% from CF-LCP, LCP-UCP, UCP-L, and L-D, respectively, for an overall increase from CF-D of 168%.

### 3.2. Reproduction

**Figure 4.**
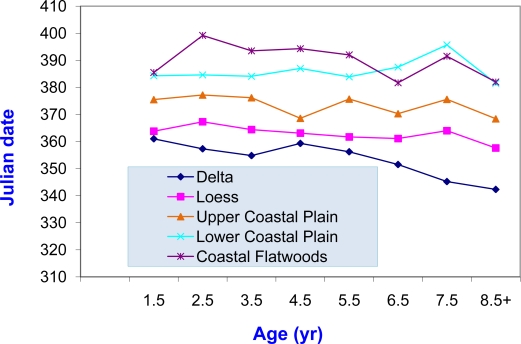
Conception dates for white-tailed deer by soils in Mississippi herd health study during March 1991–1998.

Reproduction was affected by soil region. Among adults, regional effects on fetus counts were significant (P ≤ 0.001), but not age (P = 0.20) or interactions (P = 0.11). Mean number of fetuses increased in increments of ≤ 5% (14% overall) from LCP-CF-L-D-UCP with few significant differences (P ≤ 0.05): UCP > L, CF, and LCP; D > LCP. Among adults, regional and age effects on CL were significant (P ≤ 0.002), but not interactions (P = 0.35). Mean CL increased in increments of ≤15% (29% overall) from CF, LCP, L, D, UCP with significant differences (P ≤ 0.05) being: UCP > all others, D > LCP and CF, and L > CF. Region and age affected CD significantly (P ≤ 0.001) with interactions evident (P = 0.04) which suggested that age-related development differs among regions and vice-versa. The mean CD increased between 7 and 11 days from D < L < UCP < LCP < CF (36 days overall), and each of the regions was different (P ≤ 0.05) from the other four. The CDs were the same for CF and LCP in several ages (Figure 4).

### 3.3. Discussion

A dataset of 140,276 hunter-harvested female white-tailed deer have previously been reported for Mass by soil region [8]. We analyzed the same regions and time period with different variables and found the pattern in our Mass data followed the pattern as in the larger study. Our mean Mass declined from D, L, UCP, LCP, to CF which is also the assumed order of declining soil productivity [15]. The degree of similarity between Mass in the current study and that of the larger study indicates that the current sample was a good representation of the population for condition and reproductive parameters.

In penned studies, Verme [16] reported that fawn birth weights from malnourished female deer averaged as much as 46% less than those born of properly-fed deer. Buck fawns fed poor diets in captivity weighed 50% less than controls [17]. Mass difference in our study of free-ranging doe deer of all ages from the region of least productivity was 32% less than those from the most productive soils. In contrast, enclosed fawns appeared to eat more poor forage and lost little Mass [18]. Fawns were much heavier from the prairie soil region of northern Missouri than from the remainder of the state [19]. Yearling and older male deer had markedly greater mass in the D of Mississippi [20].

The KFI serves as a condition index for free-ranging deer [21]. In our study it declined in the identical pattern by soil region as described for Mass above. Though differences in Mass are known to be influenced by heredity [22], genetics is not known to influence KFI as does stress factors such as forage quality.

Our findings were mixed, but indicate some relationship of reproductive success and soil region. Although the effects of soil region were highly significant in the present study, fetus count for CF was not different from 2 of the 3 more productive regions, and L was less than UCP. LCP did, however, have fewer CL than 2 of the best soils, and CF had fewer CL than all three of the best soils. These results indicate that maintenance of the egg and then the fetus through mid-term was not as dependent upon soil region as Mass and fat reserves, in this study. CD can be determined by aging the fetus [23] and back-dating from the date of collection. Conception progressed later in the season in perfect accord with the widely assumed declining soil productivity.

The literature was inconsistent on egg loss. Olmstead [2] found differences among soils while Rhodes *et al*. [24] reported consistency. Jones *et al*. [12,25] reported that fetal rates did not differ among the majority of soil regions and also cited McDonald [26] for fawn recruitment differences. Lactation is more indicative than other metrics of reproductive success, and the proportion of lactating adult female deer in Mississippi was lowest from the LCP and CF [25]. Penned deer were inconsistent on nutrition and conception date [27,28]. Reproductive characteristics undoubtedly vary by age of the animal due to maturity as well as by year of collection due to differing stresses from varying climate, land management practices, deer population dynamics, etc. Subsequently, manifestation of the effects of soil region may not be detectable in multi-year data sets that are lacking data for all soil regions for each year, especially where habitat quality is sufficiently high to render soil variations non-influential.

## 4. Conclusions

Our study quantified 9 years the effects of soil region on female deer KFI, fetus and CL number, and CD in addition to Mass. We know of no study to directly compare our full suite of findings, but they support the hypothesis that the relationship between Mississippi soil region and Mass and antler size would likewise be expressed in body condition and reproductive metrics of female white-tailed deer.

## Figures and Tables

**Table 1.** Body mass (Mass), kidney fat index (KFI), fetus and corpora lutea (CL) counts, and Julian conception date (CD) for female white-tailed deer from five Mississippi soils in March 1991–1998.

							Upper Coastal Plain			Lower Coastal Plain			Coastal Flatwoods		
	Delta			Loess											
	n	Mean	SD	n	Mean	SD	n	Mean	SD	n	Mean	SD	n	Mean	SD
Mass	639	41.9	7.4	774	37.3	7.6	645	34.9	5.4	258	29.3	5.7	64	28.6	6.3
(kg)											D^a^			D	
KFI	432	98	64	471	80.9	63	491	68.8	54	177	37.8	27	28	36.6	46
					B			B			C			C	
Fetus	466	1.82	0.47	540	1.77	0.47	447	1.89	0.43	177	1.66	0.53	43	1.74	0.44
		A,B			B,C			A			C			B,C	
CL	507	1.92	0.43	545	1.83	0.45	526	2.21	0.56	187	1.78	0.51	48	1.71	0.46
		B			B,C			A			C,D			D	
CD	556	356	15	630	364	15	558	375	17	206	385	13	49	392	14

a Means followed by the same letter within a row are not significantly different (P > 0.05).
